# Understanding living with tracheostomy ventilation for motor neuron disease and the implications for quality of life: a qualitative study protocol

**DOI:** 10.1136/bmjopen-2023-071624

**Published:** 2023-03-13

**Authors:** Eleanor Wilson, Nicola Turner, Christina Faull, Jonathan Palmer, Martin R Turner, Scott Davidson

**Affiliations:** 1 School of Health Sciences, University of Nottingham, Nottingham, UK; 2 LOROS Hospice, Leicester, UK; 3 Department of Thoracic Medicine, University Hospitals Plymouth NHS Trust, Plymouth, UK; 4 Nuffield Department of Clinical Neurosciences, University of Oxford, Oxford, UK; 5 Acute Services, NHS Greater Glasgow and Clyde, Glasgow, UK

**Keywords:** motor neurone disease, quality of life, respiratory medicine (see thoracic medicine), qualitative research

## Abstract

**Introduction:**

Home mechanical ventilation can be used to manage symptoms of breathlessness and sustain life for people living with motor neuron disease (plwMND). In the UK, less than 1% of plwMND use tracheostomy ventilation (TV). This contrasts with some other countries, where rates are much higher. Due to a lack of evidence about its feasibility, cost-effectiveness or outcomes, TV is not covered in the UK National Institute for Health and Care Excellence guidance. Most plwMND receiving TV in the UK do so as an unplanned crisis intervention, which can lead to a prolonged hospital stay while a complex care package is arranged. There is insufficient literature addressing the burdens and benefits of TV, how it should be initiated and delivered, and how future care choices for plwMND can be supported. The aim of this research is to provide new understandings of the experiences of plwMND using TV, and those of family members and healthcare professionals (HCPs) involved in their care.

**Methods and analysis:**

A UK-wide qualitative study with two workstreams: (1) Patient focused case studies (n=6) including plwMND, family members and HCPs to focus on experiences and tasks of daily living from multiple perspectives. (2) Interviews with plwMND (n=10), family members, including bereaved family members (n=10) and HCPs (n=20) on broader experiences and issues relating to use of TV, such as ethical considerations and decision making.

**Ethics and dissemination:**

Ethical approval has been granted by the Leicester South Research Ethics Committee (22/EM/0256). All participants will be asked to provide electronic, written and/or audio recorded informed consent. Study findings will be disseminated in peer-reviewed journals and conference presentations and used to develop new resources for teaching and public information.

**STRENGTHS AND LIMITATIONS OF THIS STUDY**

- This qualitative study will enable in-depth exploration of the views and experiences of participants on the use of tracheostomy ventilation (TV) for people living with motor neuron disease (plwMND).
- Study methods have been designed to be very flexible, enabling plwMND to participate regardless of issues associated with this progressive condition, such as communication difficulties and fatigue.
- The pool of potential participants in the UK is very small, hence it may be difficult to build diversity into study recruitment and participation in data collection.
- Participants will be self-selecting, potentially leading to recruitment bias and to some perspectives on TV being omitted from the study.

## Introduction

Motor neuron disease (MND, known as amyotrophic lateral sclerosis in its the most common form, is a progressive, neurodegenerative condition characterised by loss of motor nerves causing muscles to weaken, resulting in loss of independent limb movement, often also loss of speech and ultimately respiratory insufficiency. As respiratory muscles weaken, the National Institute for Health and Care Excellence (NICE) suggests that non-invasive ventilation (NIV) can be useful to help manage symptom burden and prolong survival.^1 2^ NIV may not be tolerated by some people living with MND (plwMND), particularly those with severe bulbar involvement, while for others, NIV might not provide sufficient ventilation as the disease progresses, nor does it protect against aspiration. Tracheostomy/laryngectomy ventilation (TV), which might support the care of such individuals, is not covered in the NICE guidance on the management of MND.^3^

Less than 1% of plwMND in the UK have TV.^4 5^ A recent audit found that TV is often undertaken in response to a crisis (81%) rather than as a planned intervention, resulting in an extended recovery time and prolonged hospital stay while a complex care package is arranged.^4^ In Italy, Spain, the USA and Japan in particular, rates of TV in plwMND are much higher (5%–33%) and increasing.^6–8^ There is evidence that TV can prolong survival times^6 7 9^ when compared with use of NIV,^8 10 11^ but evidence on quality of life for plwMND who use TV is much more limited. A small number of international studies indicate that plwMND with TV in place tend to record good quality of life scores, but family members report higher burdens of care and lower quality of life.^10 11^

In the UK, TV may be considered too intrusive and burdensome by healthcare professionals (HCPs),^12^ but there have been no substantial studies that have sought the views of plwMND or close family members on its use.^12 13^ Further, qualitative exploration would add to understanding of the lived experience of plwMND with TV, how quality of life is conceptualised in this context and what any ‘burdens’ for them or family members are. This study will build on previous work by members of the study team (JP, CF and MRT) and the UK Tracheostomy Ventilation in MND Steering Group of multidisciplinary professionals, working in association with the MND Association, to draw together evidence about the use of TV for plwMND and family members involved in their care. The findings will contribute to building an evidence base to determine how to optimise symptom management for plwMND, care pathways, choice and quality of life.

### Aims and objectives

The aims of the study are as follows:

1. To understand the experience of living with TV for plwMND, their close family members and any implications for their quality of life.
2. To understand the perspectives of HCPs on the use of TV for plwMND and any implications for treatment and care delivery pathways.

The primary objectives are as follows:

1. To explore how plwMND, family members and HCPs discuss and make decisions about the use of TV.
2. To explore the experiences of plwMND electing to have TV and those for whom it was placed in an emergency.
3. To understand the impacts and demands of living with TV from plwMND and family member perspectives.
4. To identify HCP perspectives on the key issues of TV use for plwMND and family members.

## Methods and analysis

### Study design

This is a mixed qualitative methods study comprising two workstreams:

W1: Qualitative, longitudinal case studies made up of the individual living with MND, one or more key family members directly involved in care and one or more HCP

W2: Qualitative single interviews with plwMND with TV (placed in an emergency setting or electively), close family members and a range of HCPs

W1 case studies will explore the impact of TV on day-to-day living for plwMND and those who are in regular, close contact with them. Participants will be asked to take part in an initial, qualitative interview to gather information on daily routines, experiences of care, and the benefits and challenges of living with TV. During the follow-up period of approximately 6 months, they may be asked to participate in one or more shorter interviews to deepen the enquiry into topics raised during the initial interview. PlwMND and family members may also choose to participate in taking photographs of items and spaces that illustrate daily living from their perspective, and/or creating ecograms to provide a simple visual map of their relationships and care networks.

W2 interviews will gather data on broader experiences and issues relating to the use of TV for plwMND, such as the decision-making process and patient choice. Family member participants may include bereaved family members with experience of, and insight into, these wider concerns.

### Sampling and eligibility

The study will use purposive sampling to select participants from across the UK with experience relevant to addressing the research aims. The study population will comprise adults living with MND who use TV, and adult family members and HCPs who have experience of supporting a person with MND to use TV. For case studies, the person with MND will be asked to nominate family member(s) involved in their care and HCP(s) for inclusion in the first instance. As the total number of patients with MND who use TV in the UK is small, the emphasis will be on recruiting any eligible participant, although the diversity of the sample may be limited by this approach. Inclusion and exclusion criteria are presented in box 1.

Box 1 **Inclusion and exclusion criteria**

**Inclusion criteria**

**Patient**

- Adult (aged >18 years).
- Diagnosed with motor neuron disease (MND) and using tracheostomy ventilation (TV).
- Able to use an online video conferencing tool, email or phone.
- Able to give informed consent.

**Family member**

- Adult (aged >18 years).
- In a care relationship with someone with MND who uses TV.
- Able to use an online video conferencing tool, email or phone.
- Able to give informed consent.

**Bereaved family member (workstream 2 only)**

- Adult (aged >18 years).
- Previously in a close relationship with someone with MND who uses TV.
- Bereaved >8 weeks and <3 years prior to participating in the study.
- Able to use an online video conferencing tool, email or phone.
- Able to give informed consent.

**Healthcare professional**

- Involved in the care of people with MND who use or may consider using TV.
- Able and willing to use an online video conferencing tool, email or phone.
- Able to give informed consent.

**Exclusion criteria**

**Patient**

- <18 years.
- Not diagnosed with MND.
- Not using TV.
- Unable to use an online video conferencing tool, email or phone.
- Unable to give informed consent.
- Lack or loss of capacity.

**Family member**

- <18 years.
- Not in a care relationship with someone with MND who is using TV.
- Unable to use an online video conferencing tool, email or phone.
- Unable to give informed consent.

**Bereaved family member (workstream 2 only)**

- <18 years.
- Not previously in a care relationship with someone with MND who used TV.
- Bereaved <8 weeks or >3 years prior to participating in the study.
- Unable to use an online video conferencing tool, email or phone.
- Unable to give informed consent.

**Healthcare professional**

- <18 years.
- No experience of working with plwMND.
- Unable to use an online video conferencing tool, email or phone.
- Unable to give informed consent.

### Sample size

For W1, the aim is to generate approximately six case studies, each comprising the person with MND and up to four people closely involved in their care. For W2, the aim is to conduct individual interviews with approximately 10 patients with MND with TV in place, 10 family members (including bereaved family members) and 20 HCPs across a range of professions and specialties. The estimated total sample size is 52–70 participants to provide sufficient volume and richness of data to enable a constant comparative analysis to be carried out.^14^

### Recruitment

W1: Primary participants in W1 case studies will be plwMND with elective or emergency placed TV drawn from across the UK. Patients with MND will be identified and approached through; National Health Service motor neuron disease services, palliative care or respiratory and ventilation teams, hospices, the MND association or may self-refer via public advertising. PlwMND identified by a member of their clinical team will be asked by the HCP if they are interested in taking part in the study. Patients will be given, sent or emailed an information pack comprising a letter of invitation and a participant information sheet and asked to respond directly to the researcher (by phone or email) if they are interested in taking part. Alternatively, plwMND can give their HCP permission to pass their contact details to the researcher. People who self-refer in response to public advertising will be sent or emailed an information pack and asked to respond directly to the researcher.

Potential participants will have as long as they wish to consider participation. If they wish to proceed, the researcher will make an appointment to meet with them to discuss the study, either using an online video conferencing tool (such as Teams or Zoom) or by phone. Potential participants will also have the option to liaise via email if preferred. Where it is feasible to meet in-person, and if this is the individual’s preference, the meeting will take place in a convenient location, which is likely to be their home.

Prior to the initial interview, each patient participant will be asked to nominate family member(s) and HCP(s) who have a key role in their care provision. Nominated individuals will be approached by the researcher by phone or email, provided with a participant information sheet and asked to respond directly to the researcher if they would like to take part. The researcher will arrange a convenient time to discuss the study with them and to answer any questions.

W2: PlwMND, family member and HCP participants in WS2 will be drawn from across the UK and will be identified and approached through; NHS MND services, palliative care or respiratory and ventilation teams, hospices, the MND Association or may self-refer via public advertising. In services where HCPs offer a routine postbereavement follow-up call to family members, some bereaved family members may be identified as meeting the inclusion criteria by HCPs who provided care for their relative. Bereaved family members may also be identified through bereavement services attached to the healthcare service who provided care for their relative, and with whom they have had contact since the death. HCPs may also be recruited via their clinical/professional networks such as the MND Clinical Studies Group, TV in MND Steering Group and the Specialists in Long-term Ventilation at Home network. Patients and family members can, but do not need to be from the same household. HCPs can, but do not need to be linked to a patient who is participating in the study.

PlwMND and family members identified by a member of their clinical team will be asked by the HCP if they are interested in taking part in the study. PlwMND can be given, sent or emailed an information pack comprising a letter of invitation and a participant information sheet and asked to respond directly to the researcher (by phone or email) if they are interested in taking part in the study. Alternatively, they can give their HCP permission to pass their contact details to the researcher. Those who self-refer in response to public advertising will be sent or emailed an information pack as above and asked to respond directly to the researcher.

All potential participants may take as long as they wish to consider participation. If they wish to proceed, the researcher will make an appointment to meet with them either using an online video conferencing tool, such as Teams or Zoom, or by phone. Potential participants will also have the option to liaise via email if preferred. Where it is feasible to meet in-person, and if this is the individual’s preference, the meeting will take place in a convenient location, which is likely to be their home. All potential participants will be given the opportunity to ask any questions pertaining to their involvement in the study before deciding whether to take part.

### Consent

All participants will be asked to provide electronic, written and/or audio recorded informed consent before they participate in the study. It is anticipated that electronic written consent will be the primary mode of consent. The electronic consent form will be produced using MS Forms and a secure link will be sent to each participant via email. Completed electronic consent forms will be returned directly to the researcher’s secure university account. Participants may also complete and return a consent form by post or email. Before the interview begins, the researcher and participant will go through the consent form together and verbally confirm consent, including prior electronic consent. For follow-up interviews and if a series of ongoing exchanges is used, continued consent will be checked verbally at the start of each exchange, but ongoing written responses will be considered to indicate willingness to participate.

### Data collection

Data collection will take place between January 2023 and May 2024. Interviews for WS1 and WS2 will be directed by a topic guide that can be tailored to the experiences of each participant.^15^ The topic guide for interviews with plwMND is available as an online supplemental file. Core topics have been informed by the literature and the expertise of the research team and PPI contributors. Interviews will explore decisions to have TV, whether as an elective decision or in a crisis; living with TV including family life and the care and professional input required; and consideration of deterioration, change and advance decisions. Semistructured interviews allow for core topics to be raised, while leaving scope for the identification and exploration of unanticipated topics, which may emerge as salient and extending discussion of these issues to establish clarity and depth of meaning.^14 16^

10.1136/bmjopen-2023-071624.supp1 Supplementary data

Flexibility is an essential element of this qualitative design, allowing interview techniques to be adapted to the individual. This broader concept of ‘interviewing’ recognises that interviews may take many forms including email exchanges, and every effort will be made to accommodate patients with impaired speech or fatigue due to their condition. Interviews may take place as a one-off or over several shorter exchanges.^17^ The data collection method(s) used will be guided by, and dependent on, participant’s communication preferences. PlwMND and family members participating in W1 case studies can also choose whether they prefer an individual or joint interview. All interviews will be video or audio recorded with the consent of participants and transcribed to facilitate analysis.

#### Ecograms

For W1, plwMND and family members may be asked to draw or describe services involved in providing care and support to provide a simple, visual map of their relationship networks.^18^ Ecograms allow for exploration of the range, extent and significance of those involved in supporting plwMND, including social and family contacts as well as health professionals. Previous work has shown that these can complement interviews by illustrating complexity and social burden.^19^

#### Photographs

PlwMND and family members participating in W1 may be invited to take photographs to illustrate their daily lives; for example, the spaces they inhabit, medical equipment required and things they do and enjoy. Like ecograms, photographs allow participants to express themselves in a non-verbal way, capturing the world through their eyes.^20^ Participants will be made aware that photos should not include people who have not consented to have their photo taken for the purpose of the research. Photographs may be used to inform questions posed in the interviews.^20^

Ecograms and photographs will be an optional part of the study. Where available, they may be used to prompt discussion and explore responses during case study interviews with patients and family members. The limits on the use of photographs will be clearly defined and explained to participants.

### Data analysis

As this is exploratory research, a grounded theory approach will be drawn on to implement a constant comparative analysis of the data.^14^ Analysis will be supported by NVivo V.12 software, which allows the building of individual and groups codes, helping the research team to explore how concepts are related. Open, axial and selective coding will identify core categories and thematic interrelations within and between each data set.^16^

During the initial process of open coding, segments of interview transcripts will be allocated to one or more broad ‘nodes’ within NVivo to capture all text relating to an idea or topic. The coding frame will be developed through an iterative process of reading, coding and discussion of the data by the research team to identify and link ‘themes’ occurring within and across the workstreams. Codes will remain flexible throughout so that they can be reorganised and restructured as necessary. After open coding has been completed, a more refined and selective process of coding on from individual nodes will be undertaken to differentiate and explore themes identified according to their relevance to the study objectives.

Once data collection for the case studies is complete, each case will be written up as an individual narrative, with photographs and ecograms used to generate context and perspective. Ecograms will be analysed individually and comparatively to identify patterns of key supporters within networks. Photographs will be analysed for themes across the cases as well as within each case.^20^ The visual context provided for issues raised in interviews will enable the ‘unique vitality’ of each case to be reflected.^21^

Study findings will be reported in line with Standards for Reporting Qualitative Research.^22^

### Study team

The study team is made up of clinicians, researchers and academics with expertise and specialist training in palliative care, MND and home mechanical ventilation. Team members will contribute to identifying potential participants, guiding data analysis through regular team discussions, preparing study outputs and dissemination.

### Patient and public involvement

The study patient and public involvement (PPI) group includes three plwMND and a family member currently caring for someone living with MND and using TV. The PPI group have met online and have been involved in designing the content and wording of participant information sheets, promotional materials, consent forms and topic guides. Group members will be updated on progress of the study through regular emails and virtual meetings as appropriate. The PPI group have expressed a willingness to share study information with their networks to support recruitment. They will be invited to comment on study findings and an early draft of the final report.

## Ethics and dissemination

Ethical approval was obtained from the NHS Health Research Authority in December 2022 following a favourable opinion from the Leicester South Research Ethics Committee (IRAS ID: 318973, REC Ref: 22/EM/0256). All participants will be asked to provide electronic, written and/or audio recorded informed consent prior to taking part in the study.

### Ethical considerations

The key ethical issue in this study is the sensitivity of the topic. Discussing progressive, degenerative health conditions and the end of life can be challenging for participants. Previous studies have found that those taking part in qualitative studies of this nature, including bereaved family members, report positive experiences, despite the difficulty of the subject.^23–25^ There will also be challenges in including participants with MND, as they are likely to have communication difficulties and to be easily fatigued. Yet it is vital that we do not exclude such individuals from participation in research, but tailor the research methods to meet their needs. Great care has been taken in the design of this study to allow a flexible approach to data collection based on participants’ abilities and communication preferences. Research has shown that participants who are approaching the end of life can welcome the opportunity to voice their experiences and can derive benefit from participating.^26^

Participants will be asked to consider carefully how they might feel discussing issues relating to MND and ventilation before agreeing to take part. They will be assured that participation is voluntary and that they can withdraw at any time, including during the interview. If a participant becomes distressed, the interview will be paused and time will be taken to establish whether the participant wished to continue at that time, at another time, or to withdraw from the study. The researcher will remain with the participant until they have regained composure and the discussion has been brought to a neutral topic.

Members of the research team have considerable experience of undertaking sensitive research in the field of palliative and end-of-life care and have previously worked with participants who have communication difficulties and neurological conditions. Where interviews are conducted in person the university’s lone worker policy will be followed.

### Dissemination

To maximise impact and reach, we aim to publish findings in peer-reviewed, open-access journals and to disseminate findings at national and international conferences. Other dissemination strategies will include a dedicated project website, newsletters and promotion through social media (eg, ResearchGate, Twitter, blogs). Findings will also be used to develop new resources for teaching and public information.

## Supplementary Material

Reviewer comments

Author's manuscript

## References

[1] Motor Neurone Disease Association. Ventilation for motor neurone disease. 2015. Available: www.mndassociation.org/app/uploads/2015/07/8B-Ventilation-for-motor-neurone-disease.pdf

[2] National Institute of Health and Care Excellence (NICE). Motor neurone disease: assessment and management. 2016 NICE guideline [NG42]. n.d. Available: www.nice.org.uk/guidance/NG42/chapter/Recommendations 32134605

[3] Palmer et al. 2022.

[4] Palmer J, Messer B, Ramsay M. Tracheostomy ventilation in motor neurone disease: a snapshot of UK practice. Amyotroph Lateral Scler Frontotemporal Degener 2022;23:35–41. 10.1080/21678421.2021.1916534 33969757

[5] Phelps K, Regen E, McDermott CJ, et al. Withdrawal of assisted ventilation at the patient’s request in MND/als: a retrospective exploration of the ethical and legal issues concerning relatives, nurses and allied health care professionals. Palliative Medicine [Preprint] 2022. 10.1101/2022.03.14.22271768

[6] Chiò A, Calvo A, Ghiglione P, et al. Tracheostomy in amyotrophic lateral sclerosis: a 10-year population-based study in Italy. J Neurol Neurosurg Psychiatry 2010;81:1141–3. 10.1136/jnnp.2009.175984 20660920

[7] Sancho J, Servera E, Díaz JL, et al. Home tracheotomy mechanical ventilation in patients with amyotrophic lateral sclerosis: causes, complications and 1-year survival. Thorax 2011;66:948–52. 10.1136/thx.2011.160481 21693569

[8] Tagami M, Kimura F, Nakajima H, et al. Tracheostomy and invasive ventilation in japanese ALS patients: decision-making and survival analysis: 1990-2010. J Neurol Sci 2014;344:158–64. 10.1016/j.jns.2014.06.047 25017882

[9] Phelps K, Regen E, Oliver D, et al. Withdrawal of ventilation at the patient’s Request in MND: a retrospective exploration of the ethical and legal issues that have arisen for doctors in the UK. BMJ Support Palliat Care 2017;7:189–96. 10.1136/bmjspcare-2014-000826 PMC5502251 26362794

[10] Vianello A, Arcaro G, Palmieri A, et al. Survival and quality of life after tracheostomy for acute respiratory failure in patients with amyotrophic lateral sclerosis. J Crit Care 2011;26:329. 10.1016/j.jcrc.2010.06.003 20655697

[11] Kaub-Wittemer D, Steinbüchel N von, Wasner M, et al. Quality of life and psychosocial issues in ventilated patients with amyotrophic lateral sclerosis and their caregivers. J Pain Symptom Manage 2003;26:890–6. 10.1016/s0885-3924(03)00323-3 14527757

[12] Shneerson JM. Who will benefit from tracheostomy ventilation in motor neuron disease? Thorax 2011;66:932–3. 10.1136/thoraxjnl-2011-200728 21813619

[13] Turner MR, Faull C, McDermott CJ, et al. Tracheostomy in motor neurone disease. Pract Neurol 2019;19:467–75. 10.1136/practneurol-2018-002109 31273080

[14] Charmaz K. Constructing grounded theory: a practical guide through qualitative analysis. London: Sage, 2006.

[15] Rubin H, Rubin I. Qualitative interviewing: the art of hearing data. London: Sage, 2005. 10.4135/9781452226651

[16] Strauss A, Corbin J. Basics of qualitative research, techniques and procedures for developing grounded theory. ThousandOaks: Sage Publications, 1998.

[17] Philpin SM, Jordan SE, Warring J. Giving people a voice: reflections on conducting interviews with participants experiencing communication impairment. J Adv Nurs 2005;50:299–306. 10.1111/j.1365-2648.2005.03393.x 15811109

[18] Ray RA, Street AF. Ecomapping: an innovative research tool for nurses. J Adv Nurs 2005;50:545–52. 10.1111/j.1365-2648.2005.03434.x 15882371

[19] Pollock K, Wilson E, Caswell G, et al. n.d. Family and health-care professionals managing medicines for patients with serious and terminal illness at home: a qualitative study. Health Serv Deliv Res;9:1–162. 10.3310/hsdr09140 34410684

[20] Glaw X, Inder K, Kable A, et al. Visual methodologies in qualitative research: autophotography and photo elicitation applied to mental health research. Int J Qual Methods 2017;16. 10.1177/1609406917748215 Available: 10.1177/1609406917748215

[21] Stake R. Multiple case study analysis. London: The Guilford Press, 2006.

[22] O’Brien BC, Harris IB, Beckman TJ, et al. Standards for reporting qualitative research: a synthesis of recommendations. Acad Med 2014;89:1245–51. 10.1097/ACM.0000000000000388 24979285

[23] Rosenblatt PC. Ethics of qualitative interviewing with grieving families. Death Stud 1995;19:139–55. 10.1080/07481189508252721 11652993

[24] Lowes L, Paul G. Participants’ experiences of being Interviewed about an emotive topic. J Adv Nurs 2006;55:587–95. 10.1111/j.1365-2648.2006.03950.x 16907790

[25] Cook AS. Ethical issues in bereavement research: an overview. Death Stud 1995;19:103–22. 10.1080/07481189508252719 11652991

[26] Pessin H, Galietta M, Nelson CJ, et al. Burden and benefit of psychosocial research at the end of life. J Palliat Med 2008;11:627–32. 10.1089/jpm.2007.9923 18454616 PMC3717570

