# Supplementary material for: Understanding living with tracheostomy ventilation for motor neuron disease and the implications for quality of life: a qualitative study protocol
Source: BMJ Open. 2023 Mar 13;13(3):e071624. doi: 10.1136/bmjopen-2023-071624 (PMC10016280; doi:10.1136/bmjopen-2023-071624)
Supplement: Supplementary data [file bmjopen-2023-071624supp001.pdf]

bmjopen-2023-071624 - Supplemental file

Understanding living with tracheostomy ventilation for motor neurone disease and the implications for quality of life

Person living with MND interview topic guide

In this study I am interested in the experiences of people living with MND who are using tracheostomy ventilation to support breathing, whether it was placed out of choice or following an emergency.

Just to get started can you tell me a bit about yourself?

- Age, employment, family
- Can you tell me about your illness? Date of diagnosis?
- How were you diagnosed? circumstances, symptoms, duration?
- How have things been since then?
- Who is involved in supporting you? Family/HCPs?

How did your tracheostomy ventilation come about?

- Date of trachy operation. Did you have NIV previously?
- How did you make the decision?
- Who did you discuss it with? Who else was involved? Family/HCPs?
- What information/advice were you given to help support your decision?
- Looking back, what are your thoughts about how TV came about?
- What could have been done differently to support you with this process?

If placed in an emergency

- had you expressed any wishes/discussed this option before this happened?
- What were your initial thoughts about the TV being put in place?
- What are your thoughts about it now?

What is it like to live with TV?

- How has it benefited you? What are the drawbacks?
- How has family life been affected by the TV?
- How are you supported in your daily living?
- What care package do you have in place?
- Which HCPs are involved in supporting you with your TV? What is their role?
- Have you used any type of forum/internet group or support group to discuss living with TV?
- What might you say to other people considering TV?

Wilson E, *et al. BMJ Open* 2023; 13:e071624. doi: 10.1136/bmjopen-2023-071624

What is most important to you at the moment?

- Do you have any thoughts about what might be important in the future?
- Who have you discussed this with? Family/HCPs/more widely
- Do you feel you have enough information about what might happen in the future?
- Who would you ask for more information? /discuss this with?
- Do you have any other wishes for your care in the future?
- How have you expressed these? Discussed with family / HCPs? Documented?

Is there anything about your experience of living with TV we have missed, or that you would like to add?

End of interview. Thank participant.
