# Supplementary material for: Understanding living with tracheostomy ventilation for motor neuron disease and the implications for quality of life: a qualitative study protocol
Source: BMJ Open. 2023 Mar 13;13(3):e071624. doi: 10.1136/bmjopen-2023-071624 (PMC10016280; doi:10.1136/bmjopen-2023-071624)
Supplement: Reviewer comments [file bmjopen-2023-071624.reviewer_comments.pdf]

PEER REVIEW HISTORY

BMJ Open publishes all reviews undertaken for accepted manuscripts. Reviewers are asked to complete a checklist review form (**http://bmjopen.bmj.com/site/about/resources/checklist.pdf**) and are provided with free text boxes to elaborate on their assessment. These free text comments are reproduced below.

ARTICLE DETAILS

**TITLE (PROVISIONAL)**	Understanding living with tracheostomy ventilation for motor neurone disease and the implications for quality of life: a qualitative study protocol
**AUTHORS**	Wilson, Eleanor; Turner, Nicola; Faull, Christina; Palmer, Jonathan; Turner, Martin; Davidson, Scott

This article was not externally reviewed at BMJ Open. Protocols that have been independently assessed prior to submission to BMJ Open are usually fast-tracked to publication on the grounds that further substantial changes will not be possible. This independent assessment will usually be external, independent review for both a substantial grant award from a non-commercial or government funder and ethics approval.
